# High Antenatal Psychosocial Risk Among Pregnant Women in Bulgaria: Evidence to Support Routine Mental-Health Screening

**DOI:** 10.3390/jcm14145158

**Published:** 2025-07-21

**Authors:** Elitsa Gyokova, Eleonora Hristova-Atanasova, Georgi Iskrov

**Affiliations:** 1 Department of Obstetrics and Gynecology, Faculty of Medicine, Medical University-Pleven, 5800 Pleven, Bulgaria; elitca.gaokova@mu-pleven.bg; 2 University Hospital “Saint Marina”-Pleven, 5800 Pleven, Bulgaria; 3 Department of Social Medicine and Public Health, Faculty of Public Health, Medical University of Plovdiv, 4002 Plovdiv, Bulgaria; georgi.iskrov@mu-plovdiv.bg

**Keywords:** antenatal psychosocial risk, antenatal mental health, perinatal depression, perinatal anxiety, antenatal screening, ANRQ-R, Bulgaria, Eastern Europe, maternal health, pregnancy outcomes

## Abstract

**Background:** Antenatal depression and anxiety contribute significantly to maternal morbidity and adverse pregnancy outcomes. However, structured screening and targeted interventions are largely absent from standard prenatal care in many Eastern European countries, including Bulgaria. This study examines the prevalence and psychosocial predictors of antenatal psychosocial risk using the validated Antenatal Risk Questionnaire–Revised (ANRQ-R) in a nationally underrepresented population. **Methods:** A cross-sectional survey was conducted among 216 third-trimester pregnant women in Bulgaria. Data on sociodemographic characteristics, health behaviours, and reproductive history were collected. Multivariate logistic regression identified predictors of elevated psychosocial risk. **Results:** A total of 65.7% of participants met the criteria for elevated psychosocial risk. Significant risk factors included passive smoking exposure during pregnancy (OR = 5.03, *p* < 0.001), physical activity prior to pregnancy (OR = 1.81, *p* = 0.004), and a family history of hereditary disease (OR = 42.67, *p* < 0.001). Protective factors were better self-rated current health (OR = 0.37, *p* = 0.004), the presence of chronic illness (OR = 0.42, *p* = 0.049), previous childbirth experience (OR = 0.11, *p* = 0.032), and residence in Northwestern Bulgaria (OR = 0.31, *p* = 0.028). Despite the high prevalence of psychosocial vulnerability, only 9.5% of affected women sought professional help. **Conclusions:** While our findings point to important unmet needs in antenatal mental health, further research is required before national screening policies can be implemented. Pilot programs, cultural validation of tools, and system-level readiness assessments should precede broad adoption.

## 1. Introduction

The prevalence of mental-health disorders during pregnancy and within the first year postpartum has gained significant recognition [1,2,3]. For instance, research has shown that up to 40% of pregnant women experience clinically high levels of anxiety or depressive symptoms during pregnancy [4,5].

Perinatal mental health (PMH) encompasses emotional and psychological well-being during pregnancy and the first year postpartum, including conditions such as depression and anxiety [2,3,4,5,6,7]. PMH disorders can impair maternal functioning, hinder bonding with infants, and adversely affect foetal development and child outcomes [8,9]. Symptoms often include persistent sadness, irritability, anxiety, and difficulties performing daily tasks [10,11,12,13,14,15].

The aetiology of PMH conditions is multifactorial. Research highlights numerous biological, psychological, and social risk factors, including low socioeconomic status, lack of support, history of trauma, unintended pregnancy, substance misuse, and previous mental-health disorders [16]. These findings align with the biopsychosocial and socioecological models, which underscore the dynamic interplay of individual, relational, and systemic determinants [17]. A large-scale Australian study involving over 40,000 women identified prior and current mental-health problems, domestic violence, low income, and social isolation as salient contributors to antenatal depression [16].

Over the past two decades, theoretical frameworks have increasingly acknowledged the importance of structural and policy-level interventions to support maternal mental health, especially in contexts with limited service integration. Still, major disparities persist in the availability and quality of PMH services globally [18,19,20,21]. According to the WHO, fewer than half of European countries have developed specific PMH policies or services, and routine screening is implemented in only a minority [22,23]. Against this backdrop, the present study seeks to contribute to the global and regional PMH evidence base by analysing the prevalence and predictors of antenatal psychosocial risk among pregnant women in Bulgaria, a country lacking national guidelines, structured PMH services, and routine screening programmes [22,23,24]. Existing national reports and studies reveal that PMH remains insufficiently addressed in Bulgarian clinical and academic discourse [25,26]. As Katsarova et al. note, antenatal care remains primarily somatic in focus, while emotional needs are often overlooked [27].

This study builds on socioecological and preventative mental-health frameworks and applies the Antenatal Risk Questionnaire-Revised (ANRQ-R), a validated, multidimensional screening tool that evaluates a wide spectrum of psychosocial vulnerabilities, including mental-health history, reproductive experiences, partner relationships, and social support [28]. To date, research on antenatal psychosocial risks in Bulgaria has been limited and fragmented, often lacking standardised instruments and multi-domain approaches [24,26,29].

By generating empirical evidence from a Central and Eastern European country, this study addresses a geographic gap in PMH research and policy development. It seeks to identify key demographic, behavioural, and clinical predictors of psychosocial risk and provide insights that are both nationally relevant and internationally comparable. In doing so, the research advances the field by illustrating the importance of context-sensitive screening strategies and informing the development of integrated, equitable PMH care systems.

## 2. Materials and Methods

### 2.1. Study Design

This cross-sectional study was conducted between June and October 2024 in two administrative regions of Bulgaria: the Northwestern and North-Central districts. These regions were selected to provide a demographically diverse sample, including both urban and rural populations. The study followed the STROBE (Strengthening the Reporting of Observational Studies in Epidemiology) guidelines [30] and was conducted in collaboration with the Department of Obstetrics and Gynaecology at the Medical University-Pleven and the Department of Social Medicine and Public Health at the Medical University of Plovdiv.

### 2.2. Participants and Recruitment

Pregnant women were recruited during routine third-trimester prenatal visits at the University Hospital “Saint Marina” in Pleven. The inclusion criteria were: (1) age between 18 and 49 years; (2) currently in the third trimester of pregnancy; and (3) fluency in Bulgarian. Women working in healthcare professions were excluded. This criterion was based on the assumption that healthcare professionals may have a higher awareness of mental-health issues and different response patterns, potentially skewing results.

Out of the 250 invited women, 216 met the inclusion criteria and completed the study. Only complete cases were included in the analysis. No imputation was applied for missing data. A majority of participants (55.6%) resided in the Northwestern region, while the remaining 44.4% were from the North-Central region. Recruitment was facilitated through posters in antenatal clinics and direct invitation by trained clinical staff. All participants provided electronic informed consent. Participation was voluntary, anonymous, and uncompensated.

### 2.3. Survey Instrument and ANRQ-R Implementation

Data was collected using a structured, self-administered questionnaire delivered via a tablet. The instrument was developed by the research team in accordance with national perinatal care guidelines and international standards in maternal mental-health assessment. It contained 47 close-ended items, grouped into four sections:

1. Sociodemographic information: age, ethnicity, geographic region, type of settlement (urban/rural), education level, household income, and relationship and cohabitation status;
2. Health behaviours and clinical profile: smoking and alcohol use (before and during pregnancy), physical activity, nutrition, preventive care usage, chronic and hereditary illnesses, and self-rated health status;
3. Reproductive history: number of previous births, history of spontaneous abortion, pregnancy planning, mode of delivery in prior births, and time to conception;
4. Psychosocial risk factors: evaluated using the (ANRQ-R).

We used the standard ANRQ, which comprises 12 scored items rated on a 1–5 scale (total score range 5–60), plus additional contextual questions. No modifications were made to the original instrument. A total score of ≥23 or the presence of any high-risk indicator—such as prior mental-health issues, traumatic history, or inadequate support—was used to classify respondents as being “at elevated psychosocial risk,” following established thresholds proposed by Austin et al. [28,31].

Descriptive analysis was conducted on ANRQ-R subdomains, including emotional support, trauma exposure, and anxiety traits, to identify common thematic patterns. While the total score served as the primary outcome in statistical analyses, item-level data were used for contextual interpretation. The internal consistency of the ANRQ-R in this sample was modest (Cronbach’s alpha = 0.633), which reflects its multidimensional nature but also highlights the need for further cultural validation in the Bulgarian context.

In addition to the ANRQ-R, several structured self-report questions were used to assess key psychosocial and health-related factors. Self-rated health was measured with a single-item question, a format widely recognised for its predictive validity in general and perinatal populations. Other variables—such as physical activity prior to pregnancy, exposure to passive smoking, the presence of chronic illness, and a family history of hereditary diseases—were assessed through categorical questions based on prior perinatal research. Although not standardised scales, these measures are commonly employed in maternal and public-health studies due to their practicality, interpretability, and relevance to antenatal psychosocial risk.

### 2.4. Cross-Cultural Adaptation and Pilot Testing

The ANRQ-R was translated into Bulgarian following a standardised forward–backward translation protocol as outlined by Beaton et al. [32] and Sousa and Rojjanasrirat [33]. A multidisciplinary expert team, comprising obstetricians, psychologists, and public health specialists, evaluated all items for cultural and semantic validity. Pilot testing was conducted with 10 third-trimester pregnant women. Feedback confirmed the clarity and relevance of the items, leading to minor linguistic refinements.

### 2.5. Data-Collection Procedure

Participants completed the questionnaire on-site, in a private setting, using digital tablets. A member of the research team was available for clarification but did not influence responses. To mitigate social desirability bias, no personal identifiers were recorded. The survey required approximately 20 min to complete.

### 2.6. Ethics and Participant Rights

The study was reviewed and approved by the Ethics Committee of the Medical University-Pleven (Protocol No. 737/7 June 2023). The protocol complied with the Declaration of Helsinki. The participants were informed about the study purpose, their rights to decline or withdraw at any time without consequences, and the confidentiality of their responses.

### 2.7. Statistical Analysis

Statistical analyses were conducted using IBM SPSS Statistics (version 26.0; SPSS, Inc., Chicago, IL, USA). Descriptive statistics (means, standard deviations, frequencies, percentages) were used to summarise the data. The Kolmogorov–Smirnov test assessed the normality of continuous variables. Univariate associations were explored using chi-square tests and independent-sample *t*-tests; variables with *p* < 0.10 were considered for multivariate analysis.

Binary logistic regression was used to identify predictors of elevated psychosocial risk (ANRQ-R score ≥ 23). A multivariate logistic regression model was constructed to examine the association between psychosocial risk (as measured by the ANRQ-R) and a set of independent variables. All theoretically relevant predictors and control variables were entered simultaneously in a single model. These included demographic, behavioural, clinical, and obstetric factors. Variables were retained in the model regardless of their individual statistical significance to preserve theoretical coherence and avoid omitted variable bias. Independent variables were chosen based on clinical relevance and univariate significance. Multicollinearity was assessed using the Variance Inflation Factor (VIF), with all values <2.5, indicating acceptable independence among predictors. Statistical significance was set at *p* < 0.05 (two-tailed).

Model performance was evaluated through Receiver Operating Characteristic (ROC) analysis, with area under the curve (AUC) values used to determine discrimination capacity. Model calibration was assessed via the Hosmer–Lemeshow test.

## 3. Results

### 3.1. Sociodemographic Characteristics

The total sample consisted of 216 pregnant women. The mean age was 28.3 ± 4.8 years (range: 19–42 years). The average body mass index (BMI) during pregnancy was 25.9 ± 5.5 kg/m^2^ (range: 17.8–50.1), suggesting a general tendency toward being overweight in the cohort. The vast majority of participants identified as Bulgarian (n = 212; 98.1%), while only 1.9% (n = 4) identified as Turkish. More than half of the women resided in the Northwestern region of Bulgaria (n = 120; 55.6%), and a significant proportion were living in urban areas (n = 182; 84.3%). Regarding education, 65.7% (n = 142) had completed postgraduate education; this may reflect sampling bias toward more educated women and may limit the generalisability of findings to the broader pregnant population. Regarding relationship status, 50.0% (n = 108) were cohabiting with a partner, and only 13.0% (n = 28) were married. Nearly all participants reported living with a partner or spouse (n = 210; 97.2%).

In terms of reproductive history, 62.0% (n = 134) had previously given birth, whereas 38.0% (n = 82) were nulliparous. Reported monthly household income was most commonly in the range of BGN 1000–1999 (n = 108; 50.0%) (Table 1).

### 3.2. Clinical and Behavioural Health Characteristics

In terms of lifestyle behaviours prior to pregnancy, the majority of the women (n = 180; 83.3%) reported at least occasionally following a balanced diet, and over half (n = 120; 55.6%) engaged in some form of physical activity. Notably, 51.9% (n = 112) indicated that they smoked before pregnancy, with 23.1% (n = 50) continuing to smoke during pregnancy. Additionally, 54.6% (n = 118) were exposed to secondhand smoke, either occasionally or frequently. Alcohol consumption during pregnancy was uncommon, with 91.7% (n = 198) abstaining and only 8.3% (n = 18) reporting occasional intake. Most of the women engaged in preventive healthcare, with 83.4% (n = 180) attending medical check-ups at least annually. When asked to rate their current health status, most described it as either good (n = 88; 40.7%) or very good (n = 94; 43.5%). Nearly all participants had active health insurance coverage (n = 214; 99.1%). Additionally, 86.1% (n = 186) reported no chronic health conditions, and 80.6% (n = 174) indicated no known hereditary diseases in their family. In terms of obstetric history, 12.0% (n = 26) had experienced a spontaneous abortion. Regarding delivery types, 45.6% (n = 72) had had a normal delivery, 25.3% (n = 40) a planned caesarean, and 13.9% (n = 22) had had an emergency caesarean section.

A large proportion of pregnancies were intended (n = 170; 78.7%). Among the women who had tried to conceive, 42.6% (n = 92) became pregnant within 1 to 3 months, while 36.1% (n = 78) required more than 3 months. Many women (n = 190; 88.0%) indicated that the decision to have a child was joint with their partner. The clinical and behavioural health characteristics are presented in Table 2.

### 3.3. Psychosocial and Mental-Health Factors

Further analysis of individual psychosocial and mental-health indicators revealed that 77.8% (n = 168) of the participants reported having experienced a period of two weeks or more during which they felt particularly anxious, unhappy, or depressed. Among these, 39.3% (n = 66) stated that these feelings somewhat interfered with their ability to function at work or in relationships, while 32.1% (n = 54) reported they interfered only slightly. Only 9.5% (n = 16) of those affected sought professional support. The majority consulted a general practitioner (69.0%), followed by a psychologist (21.4%), and a psychiatrist (9.5%). In total, 11.9% (n = 20) reported taking medication related to these symptoms, and 6.0% (n = 10) indicated other diagnosed mental-health problems. Regarding relational support, 44.4% (n = 96) described their current partnership as “very emotionally supportive,” and 29.6% (n = 64) as “quite a lot.” Concerning stress and life events, 68.5% (n = 148) of women reported experiencing a significant stressor, loss, or major change in the past year. Of those, 51.4% (n = 76) described the emotional impact as “quite a lot,” and 23.0% (n = 34) as “somewhat.” In terms of emotional disposition, 35.2% (n = 76) identified themselves as somewhat of a worrier, while 23.1% (n = 50) reported being “quite a lot” of a worrier. Additionally, 26.9% (n = 58) indicated they were quite affected by the absence of order in their lives, and 22.2% (n = 48) reported being “somewhat” affected. Less than half (47.2%, n = 102) felt they would not have reliable support in raising their child, while only 4.6% (n = 10) expressed high confidence in having such support. Trauma-related indicators revealed that 11.1% (n = 24) of participants had experienced emotional abuse in childhood, and 13.0% (n = 28) reported having been sexually or physically abused (Table 3).

Of the 216 pregnant women included in the study, 65.7% (n = 142) met the criteria for elevated psychosocial risk, as defined by an ANRQ-R score ≥23 or the presence of a high-risk indicator. The mean ANRQ-R score was 24.57 ± 7.60 (range 7–39).

Univariate analyses identified several sociodemographic and clinical variables significantly associated with an elevated psychosocial risk. These were subsequently included in a binary logistic regression model. The multivariate analysis revealed the following variables as significant predictors of elevated antenatal psychosocial risk.

Table 4 presents the full logistic regression model, including all predictors entered into the equation. Risk factors included passive smoking exposure during pregnancy (OR = 5.03, *p* < 0.001), engaging in physical activity before pregnancy (OR = 1.81, *p* = 0.004), and having a family history of hereditary illness (OR = 42.67, *p* < 0.001). Having had a previous birth was associated with reduced odds of elevated psychosocial risk (OR = 0.11, *p* = 0.032), suggesting a protective effect in this sample. Protective factors included better self-rated current health (OR = 0.37, *p* = 0.004), the presence of chronic illness (OR = 0.42, *p* = 0.049), and residing in certain regions (e.g., Northwestern) (OR = 0.31, *p* = 0.028). This extremely high odds ratio may indicate overfitting or the influence of unmeasured psychosocial confounders, such as perceived family burden or genetic anxiety, and should be interpreted cautiously. Non-significant variables such as smoking during pregnancy, education level, income, BMI, marital status, and alcohol use were also retained and reported for completeness.

**Figure 1:**
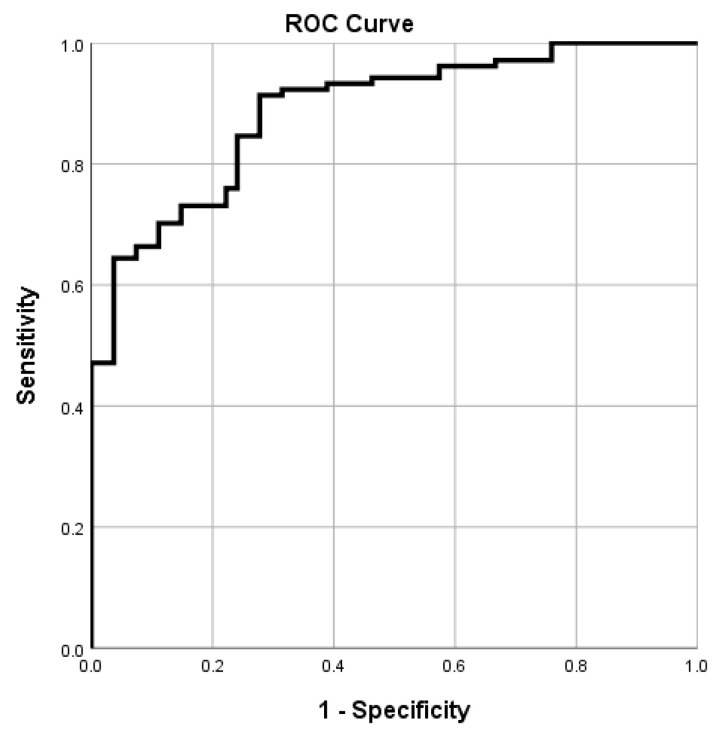
Receiver operating characteristic (ROC) curve of the binary logistic regression model for predicting elevated psychosocial risk during pregnancy.

The logistic regression model performed well overall. It demonstrated a high discriminative ability (AUC = 0.887) and correctly classified 84.8% of cases with a sensitivity of 89.4% and specificity of 75.9% (Figure 1). Model calibration was acceptable, as indicated by the Hosmer–Lemeshow test (χ^2^ = 13.21, *p* = 0.105), showing no significant discrepancy between observed and predicted values. The strength of association between predicted and actual classifications was high, with Pearson χ^2^ = 68.75 (*p* < 0.001), Likelihood Ratio χ^2^ = 70.38 (*p* < 0.001), and Cramér’s V = 0.660, suggesting strong agreement.

## 4. Discussion

### 4.1. Psychosocial Risk Prevalence and Importance 

Although PMH is widely recognised as a critical factor affecting the health of mothers and newborns [12,34], it is still not adequately addressed in many healthcare systems, including in Bulgaria. Using the ANRQ-R, this study provides new information on psychological risk during pregnancy and highlights the widespread psychosocial risk and significant systemic limitations that hinder adequate prevention, detection, and treatment of antenatal depression and anxiety.

Similar findings have been reported in multiple international studies utilising the ANRQ or its revised version (ANRQ-R). For example, Austin et al. and Reilly et al. demonstrated in Australian cohorts that the ANRQ identifies a substantial proportion of women (typically between 40% and 60%) as having an elevated psychosocial risk [35,36]. In Turkey, Cetin et al. validated the ANRQ-R and confirmed a high prevalence of psychosocial vulnerability among pregnant women, with significant unmet mental-health needs [37]. These studies reinforce the cross-cultural validity of the ANRQ-R and support its application in low-resource and transitional healthcare systems, where PMH services are still under development [35,36,37,38]. Our findings therefore align with and expand this growing body of international evidence, confirming the tool’s relevance for identifying clinically meaningful psychosocial vulnerabilities.

The study revealed that 65.7% of pregnant women scored above the threshold on the ANRQ-R, indicating elevated psychosocial vulnerability and highlighting the potential unmet mental-health needs in this population. This is a concerning figure, aligning with global estimates suggesting that perinatal depression affects approximately 10–26% of women, particularly in low- and middle-income countries [12,14,39]. 

### 4.2. Factors and Distinction from the Current Literature

Our model found several psychological vulnerability predictors. Exposure to passive smoke during pregnancy was the most significant risk factor in our model. The detrimental impact of secondhand tobacco on maternal mood and foetal outcomes is well-documented [14,40,41], and given its modifiable nature, addressing environmental tobacco exposure should be a public health priority in perinatal care.

While physical activity is typically associated with lower risk of perinatal distress, in our study it was unexpectedly linked with higher psychosocial risk. This may reflect non-recreational, work-related, or stress-compensating activity, but further data is needed to clarify the type and context of activity. This finding diverges from studies indicating that physical activity before and during pregnancy typically serves as a protective factor against perinatal depression [42]. For example, Ekelöf et al. found that women with higher levels of physical activity during pregnancy reported significantly fewer depressive symptoms postpartum, with second-trimester activity emerging as a significant predictor [43]. Similarly, Pereira et al. reported that each additional hour of pre-pregnancy physical activity reduced the odds of becoming insufficiently active during pregnancy by 14%, and that time constraints, childcare responsibilities, and work hours were major barriers to maintaining activity postpartum [44].

However, some results differ from typical expectations. Even though this correlation is statistically significant, it should be interpreted carefully. One possible explanation for our divergent findings is that the physical activity reported by participants was primarily occupational or stress-induced, rather than recreational, which may not confirm the same mental-health benefits. Alternatively, this association might reflect reverse causality, whereby women experiencing psychosocial distress engage in more physical activity as a coping mechanism. Our findings underscore the importance of differentiating between types of physical activity (occupational vs. recreational) and the psychological context in which it occurs when evaluating its relationship with PMH. Like earlier studies, a family history of hereditary (genetic) disease emerged as a substantial predictor of elevated psychosocial risk in our model. Previous research has underscored the psychological burden that awareness of genetic predispositions or familial illness may impose during pregnancy. Some studies emphasise that such awareness can increase anxiety, uncertainty about foetal health, or anticipatory stress, while others report minimal direct associations with antenatal mental-health outcomes. Notably, both genetic and family histories of mood disorders have been identified as risk factors for prenatal depression and anxiety [13,45,46]. In our study, the odds ratio associated with this variable was exceptionally high, which, although statistically significant, may reflect overfitting, sampling bias, or the influence of unmeasured psychosocial confounders. Given the wide confidence interval and the unusually strong effect size, this result should be interpreted with caution. Further validation through sensitivity analyses, stratified models, or replication in larger, more diverse populations is needed to clarify whether this relationship reflects a true causal link or a statistical artefact.

Better self-rated current health was associated with lower psychosocial risk acting as a protective factor. This association is consistent with prior research demonstrating that depressive symptoms and psychological distress during pregnancy are strongly linked to poorer self-rated health [47,48,49,50]. In this context, women with better mental well-being may also perceive and report their physical health more positively, which could explain this protective association.

The presence of chronic illness was also a protective factor; this finding contrasts with most of the literature, which suggests chronic illness increases risk. It is possible that women with chronic illness receive more frequent healthcare, thus benefitting from greater monitoring and support [39,51]. This phenomenon might be similar to research conducted in Scandinavian health systems, where integrated care strategies helped lessen the negative impact of chronic illness on mental-health outcomes [43]. However, this association should be interpreted with caution and explored in future research.

Having had a previous birth also emerged as a protective factor, potentially reflecting greater maternal experience, resilience, and confidence in managing pregnancy-related stressors. This interpretation is supported by prior findings indicating that first-time mothers may experience greater vulnerability to antenatal depression, while multiparous women often benefit from enhanced coping resources and familiarity with the pregnancy and birth process [52,53]. Additionally, residence in the Northwestern region appeared to act as a protective factor, as identified in the logistic regression model. This may be due to unmeasured contextual factors, such as closer access to university-affiliated care or stronger community ties. While this may initially seem unexpected due to socioeconomic disparities, one possible interpretation is that access to more centralised or better-coordinated obstetric and midwifery services—often concentrated around regional university hospitals may have provided improved support and continuity of care. This highlights the potential buffering role of localised, high-quality maternal health services even in demographically disadvantaged areas. To ascertain whether regional differences in psychosocial risk are a reflection of social capital, service accessibility, or other confounding factors, more research is necessary.

It is worth noting that some widely recognised predictors of PMH issues—such as low income, a single marital status, and unintended pregnancy—did not emerge as significant in this sample. This may be due to the relatively homogenous socioeconomic composition of the participants or cultural factors specific to the Bulgarian context. Similar trends have been noted in other Eastern European cohorts, where structural vulnerabilities are often counterbalanced by familial or informal support systems [54,55]. Trauma history, including emotional abuse in childhood and sexual or physical abuse, while not statistically predictive in this model, remains clinically relevant and aligns with global findings linking childhood adversity to maternal mental illness [56,57,58]. These indicators are also embedded in the ANRQ-R scoring system as individual high-risk flags and therefore play a key role in identifying women in need of closer psychosocial monitoring.

Despite such a high prevalence of psychosocial vulnerability, the study found that only 9.5% of affected women sought professional help. This may reflect pervasive stigma, insufficient mental-health literacy, and, critically, a lack of specialised services. Mental-health stigma in pregnancy remains a key barrier to care. In Bulgaria, traditional gender roles and limited awareness of perinatal disorders likely contribute to low help-seeking behaviour, as highlighted in recent studies [59,60].

### 4.3. Theoretical Implications

The findings highlight the need to understand antenatal psychosocial risk by applying socioecological and preventive frameworks. These results highlight the complex interactions between structural, cultural, relational, and personal factors, challenging linear and individualistic conceptions of vulnerability. In countries like Australia [35] and the Netherlands [55], where psychosocial screening tools like the ANRQ are incorporated into comprehensive social care systems, similar theoretical frameworks have directed national PMH initiatives. This approach is further supported by the successful adaptation of the ANRQ-R in Turkey, which shows that cultural customisation enhances clinical significance and reliability [37]. Similar to this, models such as PRAM in Canada and standard data integration systems in the UK demonstrate how screening methods gain predictive and explanatory value when they are aligned with relational and service-orientated variables [6,57,58,59,60,61]. The unexpected results regarding physical activity and chronic illnesses highlight the need to consider health behaviours and diagnoses as context-dependent variables influenced by socioeconomic status, access to healthcare, and personal experience rather than as set predictors. According to the findings, theoretical models of prenatal risk need to take this diversity into account and be empirically recalculated using datasets from a variety of cultural backgrounds, especially under-represented regions like Eastern Europe [58,59].

### 4.4. Practical Implications

The study outlines several concrete goals for Bulgaria’s maternal health policy. First of all, it highlights how urgently national PMH recommendations must be implemented in compliance with WHO and European standards [26,59]. Second, incorporating validated, culturally appropriate tools, such as the ANRQ-R, into standard prenatal care can facilitate the prompt identification of women who are at risk [55,61]. The Dutch model shows that when combined with multidisciplinary POP (Psychiatry-Obstetrics-Paediatrics) teams, referral procedures, and ongoing training, routine ANRQ-based screening may function effectively within primary-care workflows [23,56,62]. The effectiveness of ANRQ is confirmed even in systems that frequently rely on quick screening tools, as midwives report improved decision-making when it is used in conjunction with the Edinburgh Postnatal Depression Scale [45,60,63]. Third, to guarantee that screening procedures are followed, build rapport, and lessen stigma, midwives, obstetricians, and primary care physicians must be trained in psychosocial risk assessment. Even a partial implementation of PMH training improves mother disclosure and provider confidence [57,59]. Fourth, peer support groups and digital mental-health platforms are examples of community-level interventions that offer scalable and economical supplements to clinical therapy. Utilising mental-health technologies linked to screening algorithms, some nations have improved access and engagement, particularly for marginalised groups [64,65]. In a similar vein, the Indian case illustrates how digital programmes, such as the “Mother and Child Tracking System”and the use of community health workers, have successfully improved maternal mental health in environments with limited resources [66]. Resolving current barriers to access and follow-up may be made easier by integrating these tools into Bulgaria’s current maternal-care framework. Emerging projects in Eastern Europe, like mobile screening programmes and psychoeducational groups, show promise but require continued funding and evaluation [67]. Together, these global examples show that while screening instruments such as the ANRQ-R are crucial, their efficacy depends on systemic integration, professional-capacity building, and the availability of follow-up services tailored to different maternal populations.

### 4.5. Evaluation of Tools and Limitations

The ANRQ-R showed promise as a screening tool in this population. However, its modest internal consistency and lack of validation for the Bulgarian setting suggest the need for further psychometric refinement before routine clinical use [12,14,39,40,41,42,43,44]. While the internal consistency in this study was modest, this is acceptable given the multidimensional structure of the tool. The predictive model demonstrated strong discriminative ability, with acceptable calibration. Still, its ability to identify modifiable risk factors supports its inclusion in antenatal care protocols. Regular use of the ANRQ-R, combined with brief intervention strategies, could significantly improve early detection and psychosocial support [35,37].

In order to ensure the cultural and clinical relevance of the ANRQ-R for perinatal screening in Bulgaria, future studies should focus on longitudinal designs, employ a sample that is more geographically and socioeconomically diverse, incorporate qualitative methodologies, and further validate the tool.

### 4.6. Limitations

Several limitations should be considered when interpreting the findings of this study. First, the cross-sectional design precludes any conclusions about causality between the identified predictors and psychosocial risk. Second, the internal consistency of the ANRQ-R in this sample was modest (Cronbach’s alpha = 0.633), reflecting its multidimensional nature but also indicating the need for further psychometric validation in the Bulgarian context. Third, only complete cases were included in the analysis, and no imputation methods were applied, which may introduce selection bias. Fourth, the sample was geographically limited to two regions in Northern Bulgaria and predominantly included women with higher educational attainment, which may limit the generalisability of the findings to the broader national population. Fifth, the absence of a comparison group restricts the interpretability of associations and effect sizes. Sixth, while the study collected rich quantitative data, the lack of qualitative data limited our ability to explore in depth the sociocultural dimensions of stigma, mental-health literacy, and barriers to help-seeking. Seventh, interaction effects and sensitivity analyses were not conducted and should be considered in future work to improve model robustness. Finally, the lack of longitudinal follow-up means we cannot assess how antenatal psychosocial risk may evolve into postpartum psychological morbidity or may affect maternal and child outcomes over time.

Future research should prioritise longitudinal designs, include more geographically and socioeconomically diverse samples, incorporate qualitative methods, and further validate the ANRQ-R to ensure its cultural and clinical relevance for perinatal screening in Bulgaria.

## 5. Conclusions

This study provides evidence that antenatal psychosocial risk is notably prevalent among pregnant women in Bulgaria, with both behavioural and systemic factors contributing to this burden. While certain protective factors were also identified—such as better self-rated health, prior childbirth experience, and residence in the Northwestern region, the overall findings point to substantial unmet needs in PMH care. Importantly, the low rate of help-seeking behaviour among affected women highlights barriers such as stigma, limited-service availability, and possibly low mental-health literacy. The ANRQ-R showed promise as a screening instrument in this context, though its modest internal consistency suggests the need for further validation in the Bulgarian setting.

Rather than advocating for immediate national policy changes, our findings support the need for further research, including pilot screening programs, cultural adaptation and psychometric validation of the ANRQ-R, and assessment of the healthcare system’s readiness to integrate routine psychosocial screening. Ultimately, improving maternal mental health in Bulgaria will require a combination of evidence-based screening tools, provider training, targeted interventions, and public-health education tailored to the sociocultural context.

## Figures and Tables

**Table 1:** Sociodemographic characteristics of the sample.

Variable	Category	n (%)
Education	Postgraduate	142 (65.7)
	Upper secondary	74 (34.3)
Marital status	Cohabiting	108 (50.0)
	Single	80 (37.0)
	Married	28 (13.0)
Not living alone (lives with partner, family, or others)	Yes	210 (97.2)
	No	6 (2.8)
Children	0	82 (38.0)
	1	98 (45.4)
	2 or more	36 (16.7)
Income (BGN)	<999	26 (12.0)
	1000–1999	108 (50.4)
	2000–2999	56 (25.9)
	≥3000	26 (12.0)

**Table 2:** Clinical and behavioural health characteristics of the sample.

Variable	Response	n (%)
Balanced diet before pregnancy	Never	10 (4.6)
	Rarely	26 (12.0)
	Sometimes	88 (40.7)
	Usually	78 (36.1)
	Always	14 (6.5)
Physical activity before pregnancy	Never	38 (17.6)
	Rarely	58 (26.9)
	Sometimes	40 (18.5)
	Usually	46 (21.3)
	Always	34 (15.7)
Smoking before pregnancy	No	104 (48.1)
	<10 cig./day	66 (30.6)
	10–30 cig./day	46 (21.3)
Smoking during pregnancy	No	166 (76.9)
	<10 cig./day	46 (21.3)
	10–30 cig./day	4 (1.9)
Passive smoking	No	98 (45.4)
	Occasionally	96 (44.4)
	Often	22 (10.2)
Alcohol during pregnancy	No	198 (91.7)
	Occasionally	18 (8.3)
Preventive check-ups	No	14 (6.5)
	Every 6 months	82 (38.0)
	Yearly	98 (45.4)
	Every 2–3 years	22 (10.2)
Self-rated health	Bad	4 (1.9)
	Satisfactory	30 (13.9)
	Good	88 (40.7)
	Very good	94 (43.5)
Previous birth	No	82 (38.0)
	Yes	134 (62.0)
Spontaneous abortion	No	190 (88.0)
	Yes	26 (12.0)
Type of delivery	Normal	72 (45.6)
	Planned caesarean	40 (25.3)
	Emergency caesarean	22 (13.9)
Current pregnancy	Planned	170 (78.7)
	Unplanned	46 (21.3)
Time to conceive	Unplanned	46 (21.3)
	1–3 months	92 (42.6)
	3–6 months	28 (13.0)
	6–12 months	30 (13.9)
	>12 months	20 (9.2)
Reason for having a child	Partner’s request	4 (1.9)
	Own request	22 (10.2)
	Joint decision	190 (88.0)

**Table 3:** Psychosocial and mental-health indicators based on the ANRQ-R.

№	Questions	Response Distribution	ANRQ Total
Q1	Have you ever had a period of 2+ weeks when you felt anxious, unhappy or depressed?	Yes: 168 (77.8%) No: 48 (22.2%)	✔
Q1a	Did this interfere with your work or relationships? (if Q1 = Yes)	Not at all: 26 (15.5%) A little: 54 (32.1%) Somewhat: 66 (39.3%) Quite a lot: 4 (2.4%) Very much: 18 (10.7%)	✔
Q1b	Did you seek professional help? (if Q1 = Yes)	Yes: 16 (9.5%) No: 152 (90.5%)	✔
	Type of specialist consulted	GP: 116 (69.0%) Psychologist: 36 (21.4%) Psychiatrist: 16 (9.5%)	-
	Taken medication for these symptoms	Yes: 20 (11.9%) No: 148 (88.1%)	-
Q1c	Do you have/Have you had other mental-health problems?	Yes: 10 (6.0%) No: 158 (94.0%)	-
Q2	Is your relationship with your partner emotionally supportive?	Not at all: 6 (2.8%) A little: 6 (2.8%) Somewhat: 44 (20.4%) Quite a lot: 64 (29.6%) Very much: 96 (44.4%)	✔
Q3	Have you experienced stress/change/loss in the past 12 months?	Yes: 148 (68.5%) No: 68 (31.5%)	✔
Q3a	How much did/will you suffer from it? (if Q3 = Yes)	A little: 20 (13.5%) Somewhat: 34 (23.0%) Quite a lot: 76 (51.4%) Very much: 18 (12.2%)	✔
Q4	Would you describe yourself as a worrier?	Not at all: 22 (10.2%) A little: 52 (24.1%) Somewhat: 76 (35.2%) Quite a lot: 50 (23.1%) Very much: 16 (7.4%)	✔
Q5	Do you get upset if there is no order in your life?	Not at all: 42 (19.4%) A little: 48 (22.2%) Somewhat: 48 (22.2%) Quite a lot: 58 (26.9%) Very much: 20 (9.3%)	✔
Q6	Will you have people to rely on for support in raising your child?	Very much: 10 (4.6%) Quite a lot: 16 (7.4%) Somewhat: 34 (15.7%) A little: 54 (25.0%) Not at all: 102 (47.2%)	✔
Q7	Were you emotionally abused while growing up?	Yes: 24 (11.1%) No: 192 (88.9%)	✔
Q8	Have you ever been sexually or physically abused?	Yes: 28 (13.0%) No: 188 (87.0%)	✔

**Table 4:** Significant predictors of psychosocial risk.

Variable	B	OR (Exp(B))	95% CI for OR: Lower	95% CI for OR: Upper	*p*
Residence by region	−1.187	0.305	0.106	0.878	0.028
Highest education level	−0.488	0.614	0.200	1.887	0.395
Marital status	0.241	1.272	0.714	2.267	0.414
Not living alone	−20.363	0.000	0.000	—	0.999
Income	0.244	1.276	0.832	1.958	0.264
Balanced diet before pregnancy	0.182	1.199	0.611	2.353	0.597
Physical activity before pregnancy	0.593	1.810	1.209	2.710	0.004
Smoking before pregnancy	0.073	1.076	0.695	1.666	0.744
Smoking during pregnancy	0.790	2.203	0.605	8.016	0.231
Passive smoking exposure during pregnancy	1.616	5.033	2.053	12.340	0.000
Alcohol during pregnancy	−0.128	0.880	0.160	4.834	0.883
Self-rated health	−1.008	0.365	0.183	0.730	0.004
Hereditary (genetic) diseases	3.754	42.673	5.370	339.098	0.000
Chronic diseases	−0.867	0.420	0.178	0.994	0.049
Previous birth	−2.188	0.112	0.015	0.832	0.032
Type of delivery	0.324	1.383	0.877	2.179	0.163
Body mass index	−1.042	0.353	0.091	1.372	0.133

## Data Availability

Data is unavailable due to privacy or ethical restrictions.

## Abbreviations

The following abbreviations are used in this manuscript:

ANRQ-R	Antenatal Risk Questionnaire–Revised
PMH	Perinatal mental health
